# Supplementary material for: Apoptotic Quality Control of Cellular Competence Limits Cryptic Germinal Center B Cell States
Source: bioRxiv. 2026 Sep 16:2026.09.10.750694. Preprint. [Version 1] doi: 10.64898/2026.09.10.750694 (PMC13596569; doi:10.64898/2026.09.10.750694)
Supplement: Supplementary file 1 [file NIHPP2026.09.10.750694V1-supplement-1.pdf]

bioRxiv preprint doi: https://doi.org/10.64898/2026.09.10.750694; this version posted September 16, 2026. The copyright holder for this preprint (which was not certified by peer review) is the author/funder, who has granted bioRxiv a license to display the preprint in perpetuity. It is made available under a CC-BY 4.0 International license.

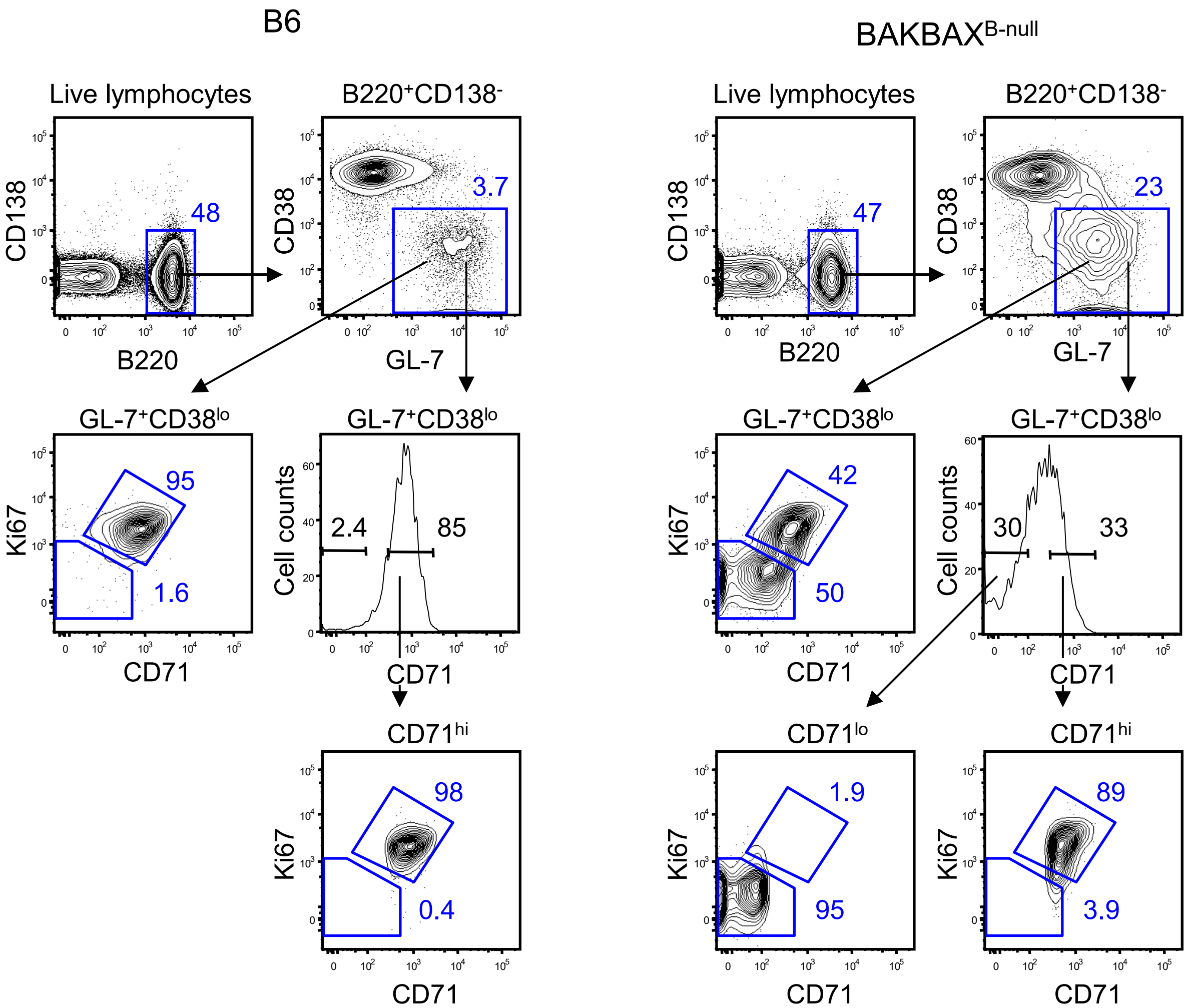

Figure S1. Association of intracellular Ki-67 and surface CD71 expressions in GC B cells

GC B cells (top panels) were further analyzed based on surface CD71 expression (x-axis) and intracellular Ki-67 expression (y-axis). Virtually all B6 GC B cells exhibited a Ki-67^{hi}CD71^{lo} phenotype, whereas BAKBAX$^{B\text{-}null}$ GC B cells contained substantial Ki-67^{hi}CD71^{hi} and Ki-67^{lo}CD71^{lo} populations (middle contour plots). Although CD71 expressions did not exhibit clearly separable peaks (middle histograms), gating the top 30% and bottom 30% of CD71-expressing cells successfully enriched Ki-67^{hi}CD71^{hi} and Ki-67^{lo}CD71^{lo} populations, respectively (bottom panels).

Fig. S1

bioRxiv preprint doi: https://doi.org/10.64898/2026.09.10.750694; this version posted September 16, 2026. The copyright holder for this preprint (which was not certified by peer review) is the author/funder, who has granted bioRxiv a license to display the preprint in perpetuity. It is made available under a CC-BY 4.0 International license.

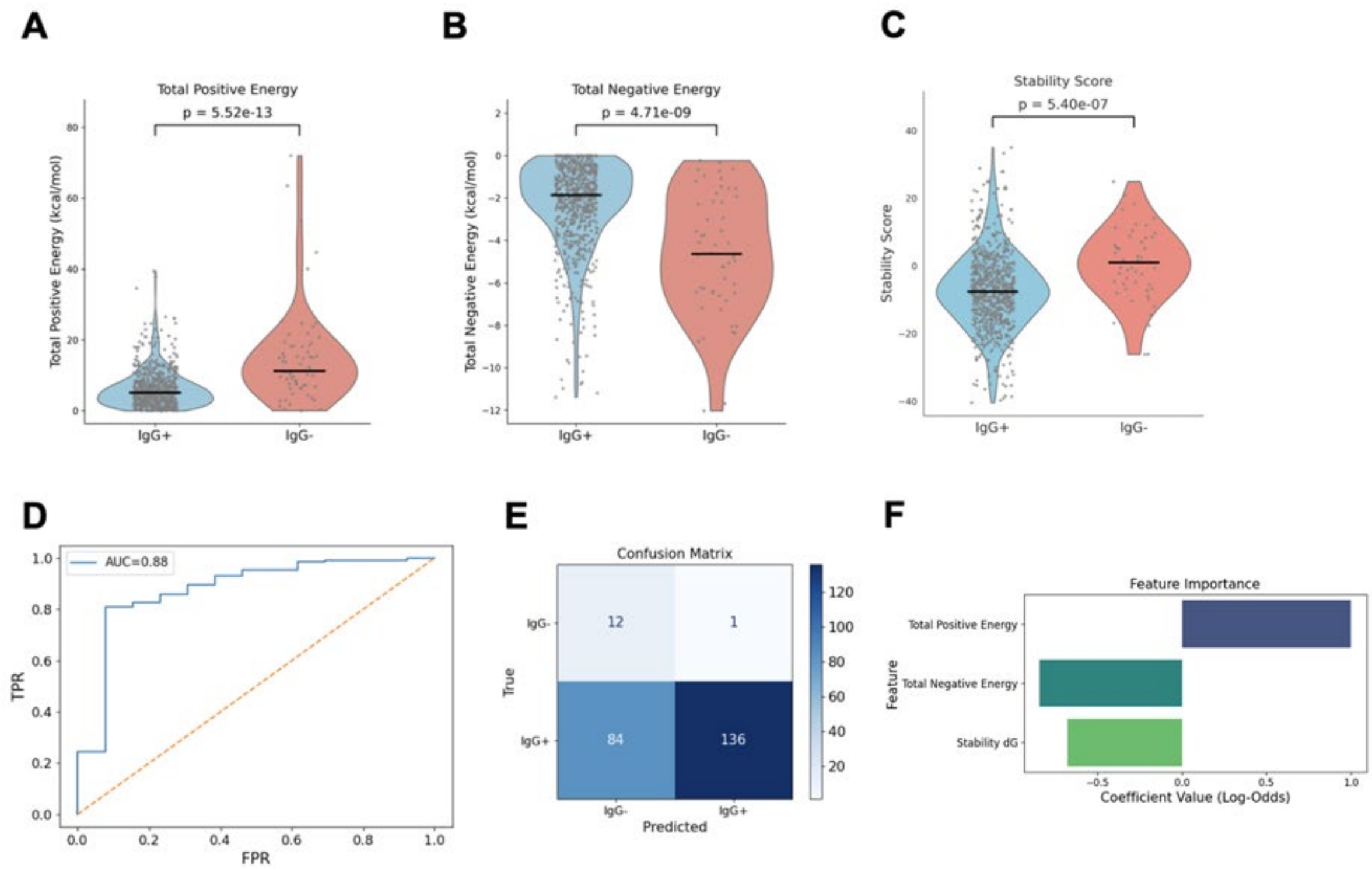

Figure S2. Modeling framework to predict IgG expression and identify candidate amino acid substitutions associated with impaired IgG expression

A logistic regression classifier was trained on features of structures predicted from antibody sequences of IgG^+ and IgG^- DZ B cells. (**A** – **C**) Distributions of feature values for IgG^+ vs. IgG^- sequences used in the model training. (**A**) Total positive energy, calculated as the sum of the FoldX estimated $\Delta\Delta G$ contributions of all predicted destabilizing mutations (*i.e.*, mutations with positive $\Delta\Delta G$ values) accumulated along the forward maturation pathway from the inferred unmutated common ancestor (UCA) to the mature antibody. Larger positive values indicate a greater cumulative destabilizing effect. (**B**) Total negative energy, calculated as the sum of the FoldX estimated $\Delta\Delta G$ contributions of all predicted stabilizing mutations (*i.e.*, mutations with negative $\Delta\Delta G$ values) accumulated along the reverse pathway from the mature antibody to the inferred UCA. More negative values indicate a greater cumulative stabilizing effect. (**C**) Stability score, calculated as the FoldX predicted structural stability dG of the mature antibody. More negative values indicate greater predicted stability. (**D**) Receiver operating characteristic (ROC) curve showing classifier performance. (**E**) Confusion matrix summarizing classification accuracy. (**F**) Feature-importance plot showing the logistic regression coefficient weights for the structural features included in the model.

bioRxiv preprint doi: https://doi.org/10.64898/2026.09.10.750694; this version posted September 16, 2026. The copyright holder for this preprint (which was not certified by peer review) is the author/funder, who has granted bioRxiv a license to display the preprint in perpetuity. It is made available under a CC-BY 4.0 International license.

Table S1. V_H gene segment usage of GC B-cell subsets

	B6 GC d8 n = 469	B6 GC d16 n = 179	B6 GC d24 n = 277	BAKBAX GC d8 n = 164	BAKBAX GC d16 n = 229	BAKBAX GC d24 n = 494	$CD71^{hi}$ DZ IgG^+ n = 476	$CD71^{lo}$ DZ IgG^+ n = 362	$CD71^{hi}$ DZ IgG^- n = 34	$CD71^{lo}$ DZ IgG^- n = 34
**1-85**	1.5	1.7	0.4	0.0	0.9	5.5	6.3	2.8	2.9	5.9
**1-84**	0.0	0.0	1.1	0.0	0.0	0.0	0.0	0.0	0.0	0.0
**1-82**	10.9	3.9	5.8	4.9	14.8	3.4	2.3	13.5	0.0	5.9
**1-81**	0.2	4.5	11.9	1.2	3.9	3.6	2.3	1.4	2.9	2.9
**1-80**	5.3	2.8	0.4	5.5	0.4	1.8	1.1	2.2	0.0	0.0
**1-78**	0.9	1.1	4.3	0.0	0.0	0.0	0.0	0.6	0.0	0.0
**1-77**	0.4	0.6	0.0	0.6	0.4	0.0	0.4	1.7	0.0	0.0
**1-76**	0.2	1.7	1.4	0.6	0.4	1.0	0.2	0.6	0.0	0.0
**1-75**	0.6	2.8	1.1	0.6	0.4	1.4	2.5	0.3	2.9	0.0
**1-74**	0.9	0.6	0.4	1.8	0.4	1.4	1.7	0.6	0.0	8.8
**1-72**	0.2	0.0	1.8	0.0	3.1	2.2	1.3	8.0	0.0	2.9
**8-12**	1.1	0.6	0.4	0.6	0.9	0.6	1.7	3.6	0.0	0.0
**1-69**	1.3	0.6	0.4	1.8	3.9	2.0	0.4	1.4	0.0	0.0
**1-67**	0.0	0.6	0.0	0.0	0.0	0.0	0.0	0.0	0.0	0.0
**1-66**	0.2	2.8	1.1	0.0	0.0	0.2	0.0	1.7	0.0	0.0
**1-64**	1.1	3.4	3.6	0.6	3.5	1.2	0.8	1.4	0.0	0.0
**1-63**	0.0	0.0	0.0	0.0	0.9	0.6	0.0	0.0	0.0	0.0
**1-62-2/1-71**	0.4	1.1	0.7	0.0	0.0	0.4	0.4	0.0	2.9	0.0
**1-61**	0.9	1.1	0.4	1.8	0.0	0.0	0.0	0.0	0.0	0.0
**1-59**	0.6	0.0	3.6	3.7	0.9	0.4	2.1	1.7	0.0	0.0
**1-58**	0.0	1.7	0.0	1.2	0.0	0.0	0.8	0.6	0.0	0.0
**8-8**	9.6	1.7	2.2	4.9	7.0	1.8	3.2	6.1	0.0	2.9
**1-55**	0.6	1.7	3.6	0.6	2.2	0.6	5.0	3.0	5.9	5.9
**1-54**	0.2	0.6	0.0	0.0	4.4	0.0	0.0	0.3	2.9	0.0
**1-53**	1.3	1.1	7.9	5.5	3.9	5.5	5.9	3.6	5.9	2.9
**1-52**	1.9	3.4	2.2	4.9	0.9	1.8	0.6	0.0	0.0	0.0
**1-50**	1.5	0.6	0.7	0.0	1.3	1.4	1.3	0.6	2.9	0.0
**1-49**	0.0	0.0	0.0	0.0	0.0	0.0	0.0	0.0	0.0	2.9
**1-47**	0.2	0.6	0.0	0.0	0.0	0.0	0.0	0.0	0.0	0.0
**1-43**	0.0	0.0	0.0	0.0	0.0	0.2	0.0	0.0	0.0	0.0
**1-42**	1.3	1.1	0.7	0.0	3.1	0.6	6.1	1.7	14.7	2.9
**1-39**	0.0	1.7	1.8	0.0	0.4	0.8	1.7	2.5	0.0	0.0
**1-36**	0.0	0.0	1.4	0.0	0.0	0.4	0.0	0.0	0.0	0.0
**1-34**	0.2	0.0	0.0	0.0	0.0	2.4	1.9	0.0	0.0	0.0
**1-31**	0.0	0.0	0.0	0.0	0.0	8.7	0.4	0.0	0.0	0.0
**1-26**	1.3	3.9	6.5	9.8	12.7	6.7	14.3	9.4	8.8	11.8
**1-22**	0.0	0.0	0.4	0.6	0.0	2.6	2.3	4.7	0.0	0.0
**1-20**	0.0	0.0	0.0	0.0	0.0	1.6	1.5	2.5	0.0	2.9
**1-19**	0.2	3.9	1.1	0.0	3.1	9.7	0.2	0.6	0.0	0.0
**1-18**	0.2	0.0	0.4	3.0	0.4	0.2	0.4	0.3	0.0	0.0
**1-15**	0.6	0.6	2.2	0.0	0.0	0.4	0.2	1.7	2.9	0.0
**1-12**	0.2	0.6	0.4	0.0	0.0	0.0	2.3	0.0	0.0	2.9
**1-11**	0.0	0.0	0.0	0.0	0.0	0.0	0.0	0.0	5.9	0.0
**1-9**	0.9	1.7	1.1	0.0	0.0	0.6	1.5	1.9	2.9	0.0
**15-2**	0.0	0.0	0.0	0.0	0.9	0.0	0.0	0.0	0.0	0.0
**1-7**	0.0	0.6	0.4	0.0	0.9	1.0	0.4	0.0	0.0	0.0
**10-3**	0.0	0.0	1.4	0.0	1.3	0.0	0.0	0.0	0.0	0.0
**1-5**	0.0	1.7	0.4	0.6	0.4	0.2	1.9	0.0	5.9	0.0
**1-4**	0.2	2.8	0.4	0.0	0.0	0.0	0.0	0.0	0.0	0.0
**10-1**	0.0	1.7	0.0	0.0	0.0	5.1	0.0	0.0	0.0	0.0
**6-6**	0.0	1.7	0.7	0.0	0.9	0.0	0.2	0.3	0.0	0.0
**6-3**	0.0	0.6	1.4	0.6	0.0	1.2	0.2	0.0	2.9	0.0
**3-8**	0.0	0.0	0.7	0.0	0.0	0.4	0.0	0.0	0.0	0.0
**9-4**	0.0	0.6	0.0	0.0	0.0	0.6	3.2	0.3	2.9	5.9
**3-6**	0.9	1.1	4.0	0.0	1.7	1.2	1.9	0.3	0.0	2.9
**3-4**	0.2	0.0	0.4	0.0	0.0	0.0	0.0	0.0	0.0	0.0
**14-4**	40.7	0.6	2.5	34.1	3.1	1.0	1.3	6.9	0.0	14.7
**7-3**	0.2	1.1	0.0	0.0	0.0	0.2	1.1	0.3	0.0	0.0
**9-3**	0.4	2.2	1.1	0.6	4.8	0.4	1.5	3.6	5.9	14.7
**9-1**	1.7	0.0	0.4	0.6	0.0	0.4	0.2	0.3	2.9	0.0
**14-3**	0.0	1.7	0.4	0.0	3.9	7.5	0.2	0.0	0.0	0.0
**14-2**	3.4	19.0	4.0	0.6	0.9	3.2	5.7	1.4	2.9	0.0
**3-1**	0.2	0.6	0.0	0.0	0.4	0.4	0.0	1.1	0.0	0.0
**4-1**	0.2	0.0	0.0	0.0	0.0	0.0	0.2	0.0	0.0	0.0
**14-1**	3.0	1.7	1.1	3.0	0.9	0.6	0.2	0.8	2.9	0.0
**2-9**	0.2	0.0	0.0	1.8	0.0	0.0	0.0	0.0	0.0	0.0
**5-17**	0.2	1.1	0.7	0.0	1.3	0.8	0.4	1.4	0.0	0.0
**5-16**	0.2	0.6	0.0	0.0	0.0	0.0	0.4	0.6	0.0	0.0
**2-9-1**	0.2	0.0	0.0	0.0	0.0	0.0	0.0	0.3	0.0	0.0
**5-9-1**	0.2	0.6	0.0	0.0	0.0	0.0	0.0	0.3	0.0	0.0
**2-6**	0.0	0.0	0.4	1.2	0.0	0.2	5.9	0.3	5.9	0.0
**5-12**	0.0	0.6	0.0	0.0	0.0	0.0	0.2	0.0	0.0	0.0
**2-5**	0.0	1.1	0.0	0.0	0.9	0.0	0.0	0.0	0.0	0.0
**2-4**	0.0	0.0	0.0	1.2	0.0	0.0	0.0	0.0	0.0	0.0
**5-6**	0.4	3.9	5.4	0.6	2.6	2.6	1.3	0.8	2.9	0.0
**2-3**	0.0	0.0	0.4	0.0	0.0	0.0	0.0	0.6	0.0	0.0
**5-4**	0.2	0.6	0.4	0.6	0.9	0.0	0.6	0.0	0.0	0.0
**2-2**	0.2	0.0	2.5	0.0	0.0	0.8	0.0	0.3	0.0	0.0

Table S1

The percentages of V_H gene segments among all V_H gene segments within each B-cell subset are shown.

bioRxiv preprint doi: https://doi.org/10.64898/2026.09.10.750694; this version posted September 16, 2026. The copyright holder for this preprint (which was not certified by peer review) is the author/funder, who has granted bioRxiv a license to display the preprint in perpetuity. It is made available under a CC-BY 4.0 International license.

Table S2. Number of clonal cultures with or without IgG[1]

GC B cells	IgG^{+}	IgG^{-}	Ratio (IgG^{-}/IgG^{+})
$BAKBAX^{B\text{-}null}$ $CD71^{hi}$ DZ[2]	208	64	0.31
$BAKBAX^{B\text{-}null}$ $CD71^{lo}$ DZ[2]	146	51	0.35
B6 GC DZ[2]	54	1	0.02
B6 GC[3]	129	0	0.00

Table S2

[1] On days 5-8 of Nojima cultures, presence of expanded single B cells was determined using light microscopy. After Nojima cultures, IgG secretion was determined by ELISA. IgG^{+} represents clonal cultures that contain IgG. IgG^{-} represents clonal cultures that contain expanded B cells without secreting IgG.

[2] DZ B cells were sorted from popliteal LNs of B6 or $BAKBAX^{B\text{-}null}$ mice following footpad immunization with rPA in alhydrogel.

[3] GC B cells were sorted from inguinal LNs of B6 mice following s.c. immunization with ∆gD-2 herpes virus vaccine candidate.

bioRxiv preprint doi: https://doi.org/10.64898/2026.09.10.750694; this version posted September 16, 2026. The copyright holder for this preprint (which was not certified by peer review) is the author/funder, who has granted bioRxiv a license to display the preprint in perpetuity. It is made available under a CC-BY 4.0 International license.

Table S3. Number of Replacement and Silent mutations in V_H and $V_{K/L}$ genes

DZ subset	V_H CDR				V_H FR				$V_{K/L}$ CDR				$V_{K/L}$ FR			
	R	S	R/S		R	S	R/S		R	S	R/S		R	S	R/S	
$CD71^{hi}IgG^-$	37	6	6.2		88	22	4.0		16	1	16		61	15	4.1	
$CD71^{lo}IgG^-$	19	5	3.8	n.s.	82	22	3.7	**	8	0	N/A	n.s.	72	29	2.5	n.s.
IgG^- total	56	11	5.1		170	44	3.9		24	1	24		133	44	3.0	
$CD71^{hi}IgG^+$	434	77	5.6		934	399	2.3		192	31	6.2		802	307	2.6	
$CD71^{lo}IgG^+$	334	50	6.7		724	285	2.5		162	38	4.3		603	206	2.9	
IgG^+ total	768	127	6.0		1658	684	2.4		354	69	5.1		1405	513	2.7	

Table S3
After Nojima culture of $CD71^{hi}$ or $CD71^{lo}$ DZ cells, V(D)J gene rearrangements for heavy- and light-chains were amplified by RT-PCR (see the legends of **Fig. 6** and **Fig. 7**). Numbers of replacement (R) mutations and Silent mutations (S), and R to S ratio (R/S) are shown. CDR, CDR1 and CDR2; FR, Framework regions 1 – 3. n.s., $p > 0.05$; **, $p < 0.01$ determined by Chi-square test with Yates' correction.

bioRxiv preprint doi: https://doi.org/10.64898/2026.09.10.750694; this version posted September 16, 2026. The copyright holder for this preprint (which was not certified by peer review) is the author/funder, who has granted bioRxiv a license to display the preprint in perpetuity. It is made available under a CC-BY 4.0 International license.

Table S4. Primer sequences

RT for RT-PCR A	Name	Sequence (5' → 3')
SMART template-switching oligo[a,b]	TSO-bioG	/5Biosg/CCAAGCTGGCTAGCACCATGACAGrGrGrG
SMART template-switching oligo[a,b]	TSO-P1	/5Biosg/CCAAGCTGGCTAGCACCATGACAGgtagcactCAGrGrGrG
SMART template-switching oligo[a,b]	TSO-P2	/5Biosg/CCAAGCTGGCTAGCACCATGACAGtcagtcgaCAGrGrGrG
SMART template-switching oligo[a,b]	TSO-P3	/5Biosg/CCAAGCTGGCTAGCACCATGACAGgcatgtacCAGrGrGrG
SMART template-switching oligo[a,b]	TSO-P4	/5Biosg/CCAAGCTGGCTAGCACCATGACAGgcgtacatCAGrGrGrG
SMART template-switching oligo[a,b]	TSO-P5	/5Biosg/CCAAGCTGGCTAGCACCATGACAGgtgacatcCAGrGrGrG
SMART template-switching oligo[a,b]	TSO-P6	/5Biosg/CCAAGCTGGCTAGCACCATGACAGctgactagCAGrGrGrG
SMART template-switching oligo[a,b]	TSO-P7	/5Biosg/CCAAGCTGGCTAGCACCATGACAGtatcgcagCAGrGrGrG
SMART template-switching oligo[a,b]	TSO-P8	/5Biosg/CCAAGCTGGCTAGCACCATGACAGcgctatgaCAGrGrGrG
SMART template-switching oligo[a,b]	TSO-P9	/5Biosg/CCAAGCTGGCTAGCACCATGACAGagtcagctCAGrGrGrG
SMART template-switching oligo[a,b]	TSO-P10	/5Biosg/CCAAGCTGGCTAGCACCATGACAGcgtacatgCAGrGrGrG
SMART template-switching oligo[a,b]	TSO-P11	/5Biosg/CCAAGCTGGCTAGCACCATGACAGtacgtgcaCAGrGrGrG
SMART template-switching oligo[a,b]	TSO-P12	/5Biosg/CCAAGCTGGCTAGCACCATGACAGcatgcgtaCAGrGrGrG
gene-specific reverse primers	mIgG-RV1	GAGATGGTTYTCTCGATGGG
gene-specific reverse primers	mIgM-RV1	GGAATGGGCACATGCAGA
gene-specific reverse primers	mIgK-RV1	CTAACACTCATTCCTGTTGAAG
gene-specific reverse primers	mIgL23-RV1	AGACATTCTGCAGGAGACA
gene-specific reverse primers	mIgL145-RV1	AACASTCAGCACGGGACA

Primary PCR for RT-PCR A	Name	Sequence (5' → 3')
Forward primer[c]	5Anchor-1	GGTAGatagcgtcCAAGCTGGCTAGCACCATGA
Forward primer[c]	5Anchor-2	GGTAGacagtgctCAAGCTGGCTAGCACCATGA
Forward primer[c]	5Anchor-3	GGTAGgtacgcatCAAGCTGGCTAGCACCATGA
Forward primer[c]	5Anchor-4	GGTAGgtgcatacCAAGCTGGCTAGCACCATGA
Forward primer[c]	5Anchor-5	GGTAGacgatgtcCAAGCTGGCTAGCACCATGA
Forward primer[c]	5Anchor-6	GGTAGtcgatcagCAAGCTGGCTAGCACCATGA
Forward primer[c]	5Anchor-7	GGTAGgactgatcCAAGCTGGCTAGCACCATGA
Forward primer[c]	5Anchor-8	GGTAGcgtcatagCAAGCTGGCTAGCACCATGA
Forward primer[c]	5Anchor-9	GGTAGcatcgtgaCAAGCTGGCTAGCACCATGA
Forward primer[c]	5Anchor-10	GGTAGagctagtcCAAGCTGGCTAGCACCATGA
Forward primer[c]	5Anchor-11	GGTAGcacgtatgCAAGCTGGCTAGCACCATGA
Forward primer[c]	5Anchor-12	GGTAGtatgcacgCAAGCTGGCTAGCACCATGA
Recverse primer for IgH	mIgG-RV2	ACCYTGCATTTGAACTCCTTG

Secondary PCR for RT-PCR A	Name	Sequence (5' → 3')
Forward primer[c]	5Anchor-1	GGTAGatagcgtcCAAGCTGGCTAGCACCATGA
Forward primer[c]	5Anchor-2	GGTAGacagtgctCAAGCTGGCTAGCACCATGA
Forward primer[c]	5Anchor-3	GGTAGgtacgcatCAAGCTGGCTAGCACCATGA
Forward primer[c]	5Anchor-4	GGTAGgtgcatacCAAGCTGGCTAGCACCATGA
Forward primer[c]	5Anchor-5	GGTAGacgatgtcCAAGCTGGCTAGCACCATGA
Forward primer[c]	5Anchor-6	GGTAGtcgatcagCAAGCTGGCTAGCACCATGA
Forward primer[c]	5Anchor-7	GGTAGgactgatcCAAGCTGGCTAGCACCATGA
Forward primer[c]	5Anchor-8	GGTAGcgtcatagCAAGCTGGCTAGCACCATGA
Forward primer[c]	5Anchor-9	GGTAGcatcgtgaCAAGCTGGCTAGCACCATGA
Forward primer[c]	5Anchor-10	GGTAGagctagtcCAAGCTGGCTAGCACCATGA
Forward primer[c]	5Anchor-11	GGTAGcacgtatgCAAGCTGGCTAGCACCATGA
Forward primer[c]	5Anchor-12	GGTAGtatgcacgCAAGCTGGCTAGCACCATGA
Reverse primer for IgH[d]	mIgM-A	GGTAGgatactgcGAGGTGGCTAGGTASTTGCC
Reverse primer for IgH[d]	mIgM-B	GGTAGacgcatgtGAGGTGGCTAGGTASTTGCC
Reverse primer for IgH[d]	mIgM-C	GGTAGtcagctagGAGGTGGCTAGGTASTTGCC
Reverse primer for IgH[d]	mIgM-D	GGTAGagtcgatcGAGGTGGCTAGGTASTTGCC
Reverse primer for IgH[d]	mIgM-E	GGTAGctgtagcaGAGGTGGCTAGGTASTTGCC
Reverse primer for IgH[d]	mIgM-F	GGTAGtcgcagtaGAGGTGGCTAGGTASTTGCC
Reverse primer for IgH[d]	mIgM-G	GGTAGtacatgcgGAGGTGGCTAGGTASTTGCC
Reverse primer for IgH[d]	mIgM-H	GGTAGcgtatgcaGAGGTGGCTAGGTASTTGCC
Reverse primer for IgH[d]	mIgG12b-A	GGTAGgatactgcGGGGACAGTCACTGAGCTG
Reverse primer for IgH[d]	mIgG12b-B	GGTAGacgcatgtGGGGACAGTCACTGAGCTG
Reverse primer for IgH[d]	mIgG12b-C	GGTAGtcagctagGGGGACAGTCACTGAGCTG
Reverse primer for IgH[d]	mIgG12b-D	GGTAGagtcgatcGGGGACAGTCACTGAGCTG
Reverse primer for IgH[d]	mIgG12b-E	GGTAGctgtagcaGGGGACAGTCACTGAGCTG
Reverse primer for IgH[d]	mIgG12b-F	GGTAGtcgcagtaGGGGACAGTCACTGAGCTG
Reverse primer for IgH[d]	mIgG12b-G	GGTAGtacatgcgGGGGACAGTCACTGAGCTG
Reverse primer for IgH[d]	mIgG12b-H	GGTAGcgtatgcaGGGGACAGTCACTGAGCTG
Reverse primer for IgH[d]	mIgG2ac-A	GGTAGgatactgcGAGGTTACAGTCACTGAGCTG
Reverse primer for IgH[d]	mIgG2ac-B	GGTAGacgcatgtGAGGTTACAGTCACTGAGCTG
Reverse primer for IgH[d]	mIgG2ac-C	GGTAGtcagctagGAGGTTACAGTCACTGAGCTG
Reverse primer for IgH[d]	mIgG2ac-D	GGTAGagtcgatcGAGGTTACAGTCACTGAGCTG
Reverse primer for IgH[d]	mIgG2ac-E	GGTAGctgtagcaGAGGTTACAGTCACTGAGCTG
Reverse primer for IgH[d]	mIgG2ac-F	GGTAGtcgcagtaGAGGTTACAGTCACTGAGCTG
Reverse primer for IgH[d]	mIgG2ac-G	GGTAGtacatgcgGAGGTTACAGTCACTGAGCTG
Reverse primer for IgH[d]	mIgG2ac-H	GGTAGcgtatgcaGAGGTTACAGTCACTGAGCTG
Reverse primer for IgH[d]	mIgG3-A	GGTAGgatactgcGGGTACAGTCACCAAGCTG
Reverse primer for IgH[d]	mIgG3-B	GGTAGacgcatgtGGGTACAGTCACCAAGCTG
Reverse primer for IgH[d]	mIgG3-C	GGTAGtcagctagGGGTACAGTCACCAAGCTG
Reverse primer for IgH[d]	mIgG3-D	GGTAGagtcgatcGGGTACAGTCACCAAGCTG
Reverse primer for IgH[d]	mIgG3-E	GGTAGctgtagcaGGGTACAGTCACCAAGCTG
Reverse primer for IgH[d]	mIgG3-F	GGTAGtcgcagtaGGGTACAGTCACCAAGCTG
Reverse primer for IgH[d]	mIgG3-G	GGTAGtacatgcgGGGTACAGTCACCAAGCTG
Reverse primer for IgH[d]	mIgG3-H	GGTAGcgtatgcaGGGTACAGTCACCAAGCTG

[a]/5Biosg/ represents a biotin at 5' end. There are three riboguanosines (rGrGrG) at 3' end.
[b]These primers contain plate-associated barcodes (0-12) that are indicated in lowercase letters.
[c]These primers contain well-associated barcodes (1-12) that are indicated in lowercase letters.
[d]These primers contain well-associated barcodes (A-H) that are indicated in lowercase letters.

RT for RT-PCR B	Name	Sequence (5' → 3')
gene-specific reverse primers	mIgG-RV1	GAGATGGTTYTCTCGATGGG
gene-specific reverse primers	mIgKC2-RV1	CAGGACCTTTGTCTCTAACAC
gene-specific reverse primers	mIgL23-RT	GTTCCYAGGCTAAGGAGAAAC
gene-specific reverse primers	mIgLC1-RT	CCTCTGTGGGTAAAATGAAG
gene-specific reverse primers	mIgLC4-RT	CCTCTGTGGGTAAGGTGAG

Primary PCR for RT-PCR B	Name	Sequence (5' → 3')
Forward primer	mVH1-1	agRtYcagctgcaRcagtct
Forward primer	mVH1-2	aggtccaactgcagcagcc
Forward primer	mVH2	tctgcctggtgacWttccca
Forward primer	mVH3	gtgcagcttcaggagtcag
Forward primer	mVH4	gaggtgaagcttctcgagtc
Forward primer	mVH5	gaagtgaagctggtggagtc
Forward primer	mVH6	atgKacttgggactgaRctgt
Forward primer	mVH7	cagtgtgaggtgaagctggt
Forward primer	mVH8	ccaggttactctgaaagagtc
Forward primer	mVH9	tgtggaccttgctattcctga
Forward primer	mVH10	tgttggggctgaagtgggttt
Forward primer	mVH11	atggagtgggaactgagctta
Forward primer	mVH12	agcttcaggagtcaggacc
Forward primer	mVH13	caggtgcagcttgtagagac
Forward primer	mVH14	atgcagctgggtcatcttctt
Forward primer	mVH15	gactggatttggatcacKctc
Forward primer	mVH16	tggagtttggacttagttggg
Recverse primer for IgH	mIgHGC-RV2	GTTGACCYTGCATTTGAACTC

Secondary PCR for RT-PCR B	Name	Sequence (5' → 3')
Forward primer[c]	mIgHV-FW-1	GGTAGatagcgtcGAGGTGCAGCTGCAGGAGTCTGG
Forward primer[c]	mIgHV-FW-2	GGTAGacagtgctGAGGTGCAGCTGCAGGAGTCTGG
Forward primer[c]	mIgHV-FW-3	GGTAGgtacgcatGAGGTGCAGCTGCAGGAGTCTGG
Forward primer[c]	mIgHV-FW-4	GGTAGgtgcatacGAGGTGCAGCTGCAGGAGTCTGG
Forward primer[c]	mIgHV-FW-5	GGTAGacgatgtcGAGGTGCAGCTGCAGGAGTCTGG
Forward primer[c]	mIgHV-FW-6	GGTAGtcgatcagGAGGTGCAGCTGCAGGAGTCTGG
Forward primer[c]	mIgHV-FW-7	GGTAGgactgatcGAGGTGCAGCTGCAGGAGTCTGG
Forward primer[c]	mIgHV-FW-8	GGTAGcgtcatagGAGGTGCAGCTGCAGGAGTCTGG
Forward primer[c]	mIgHV-FW-9	GGTAGcatcgtgaGAGGTGCAGCTGCAGGAGTCTGG
Forward primer[c]	mIgHV-FW-10	GGTAGagctagtcGAGGTGCAGCTGCAGGAGTCTGG
Forward primer[c]	mIgHV-FW-11	GGTAGcacgtatgGAGGTGCAGCTGCAGGAGTCTGG
Forward primer[c]	mIgHV-FW-12	GGTAGtatgcacgGAGGTGCAGCTGCAGGAGTCTGG
Reverse primer for IgH[d]	mIgHC12b-RV-A	GGTAGgcagtatcCGYTGCAGGTGACGGTCTG
Reverse primer for IgH[d]	mIgHC12b-RV-B	GGTAGacgcatgtCGYTGCAGGTGACGGTCTG
Reverse primer for IgH[d]	mIgHC12b-RV-C	GGTAGagcgtcatCGYTGCAGGTGACGGTCTG
Reverse primer for IgH[d]	mIgHC12b-RV-D	GGTAGagtcgatcCGYTGCAGGTGACGGTCTG
Reverse primer for IgH[d]	mIgHC12b-RV-E	GGTAGctgtagcaCGYTGCAGGTGACGGTCTG
Reverse primer for IgH[d]	mIgHC12b-RV-F	GGTAGtcgcagtaCGYTGCAGGTGACGGTCTG
Reverse primer for IgH[d]	mIgHC12b-RV-G	GGTAGcgcatgtaCGYTGCAGGTGACGGTCTG
Reverse primer for IgH[d]	mIgHC12b-RV-H	GGTAGcgtatgcaCGYTGCAGGTGACGGTCTG
Reverse primer for IgH[d]	mIgHC2ac-RV-A	GGTAGgcagtatcACATTGCAGGTGATGGWCTG
Reverse primer for IgH[d]	mIgHC2ac-RV-B	GGTAGacgcatgtACATTGCAGGTGATGGWCTG
Reverse primer for IgH[d]	mIgHC2ac-RV-C	GGTAGagcgtcatACATTGCAGGTGATGGWCTG
Reverse primer for IgH[d]	mIgHC2ac-RV-D	GGTAGagtcgatcACATTGCAGGTGATGGWCTG
Reverse primer for IgH[d]	mIgHC2ac-RV-E	GGTAGctgtagcaACATTGCAGGTGATGGWCTG
Reverse primer for IgH[d]	mIgHC2ac-RV-F	GGTAGtcgcagtaACATTGCAGGTGATGGWCTG
Reverse primer for IgH[d]	mIgHC2ac-RV-G	GGTAGcgcatgtaACATTGCAGGTGATGGWCTG
Reverse primer for IgH[d]	mIgHC2ac-RV-H	GGTAGcgtatgcaACATTGCAGGTGATGGWCTG

[c]These primers contain well-associated barcodes (1-12) that are indicated in lowercase letters.
[d]These primers contain well-associated barcodes (A-H) that are indicated in lowercase letters.
